# Topological insulator with negative spin-orbit coupling and transition between Weyl and Dirac semimetals in InGaN-based quantum wells

**DOI:** 10.1038/s41598-018-33461-4

**Published:** 2018-10-18

**Authors:** S. P. Łepkowski, W. Bardyszewski

**Affiliations:** 1 Institute of High Pressure Physics,”Unipress”, Polish Academy of Sciences, ul. Sokołowska 29/37, 01-142 Warszawa, Poland; 2 Faculty of Physics, University of Warsaw, ul. Pasteura 5, 02-093 Warszawa, Poland

## Abstract

We study the influence of negative spin-orbit coupling on the topological phase transition and properties of the topological insulator state in InGaN-based quantum wells grown along *c* axis of the wurtzite lattice. The realistic eight-band *k·p* method with relativistic and nonrelativistic linear-*k* terms is employed. Our calculations show that the negative spin-orbit coupling in InN is not an obstacle to obtain the topological insulator phase in InN/InGaN and InGaN/GaN quantum wells. The bulk energy gap in the topological insulator state can reach 2 meV, which allows experimental verification of the edge state transport in these materials. The topological phase transition occurs due to the band inversion between the highest light hole subband and the lowest conduction subband, and almost always is mediated by the two-dimensional Weyl semimetal, arising from an anticrossing of these subbands at zero in-plane wave vector. However, for certain InGaN/GaN quantum wells, we find that the magnitude of this anticrossing vanishes, leading to the appearance of the Dirac semimetal. The novel transition between the Weyl and Dirac semimetals originates from vanishing of the average in-plane spin-orbit interaction parameter, which decouples the conduction subband from the light hole subband at zero in-plane wave vector.

## Introduction

The discovery of the time–reversal topological insulators (TIs) in two and three dimensions has greatly inspired the study of topological properties of the electronic band structure of crystalline materials^1^. The TIs are characterized by an energy gap in the bulk electronic band structure and metallic states on the boundaries. Closing of the band gap by the surface or edge states is caused by the nontrivial topology of the bulk states, originating from an inversion in the order in the valence and conduction bands at time reversal invariant wave vectors in the Brillouin zone (BZ)^1^. This band inversion changes the Z_2_ topological invariant leading to the topological phase transition (TPT) from the normal insulator (NI) to the TI state^1^. The nature of the TPT depends on the dimensionality and crystal symmetry of the systems^2–11^. In three-dimensional (3D) crystals without inversion symmetry, the TPT is mediated either by a stable Weyl semimetal (WSM) phase with separated Weyl points or a nodal-line semimetal having a line nodes along which the band gap closes^2,3^. When the system has inversion symmetry, a direct transition between the NI and TI phases occurs through a critical point corresponding to a Dirac semimetal (DSM)^2,3^. The DSMs arising in the TPT are generally not robust against small perturbations and in certain cases they can be stabilized by the crystal symmetry. The symmetry protected Dirac states occur at high-symmetry points on the surface of the BZ in crystals with the nonsymmorphic space group symmetries or at generic points on a n-fold symmetry axis inside the BZ, where the mixing between the inverted bands is forbidden by the different rotational symmetries^4,5^. Recently, it has also been found that in the 3D systems, it is possible to induce the TPT without closing of the band gap, which can happen due to a jump between two band gap minima in the free energy corresponding to the NI and TI states^6^.

In two-dimensional (2D) nanostructures, the TPT is always accompanied by the closing of the bulk band gap, and the character of the intermediate gapless states depends on the full crystal symmetry of the multilayer structures^6–11^. In conventional 2D topological materials, such as zinc-blende HgTe/CdTe and InAs/GaSb/AlSb quantum wells (QWs), the band inversion occurs in the center of the BZ, and the TPT is mediated either by the DSM or by the WSM, depending whether the conduction band (CB) and the valence band (VB) states cross or anticross at zero in-plane wave vector (*k*_⊥_ = 0), respectively^8–10^. When the QW structure is oriented along [001] crystallographic direction, both the CB and heavy hole (HH) states transform according to the same spinor representations, and thus, they anticross at *k*_⊥_ = 0, generating the WSM at the boundary between the NI and TI states^8–10^. Recently, it has been shown that the WSM in these nanostructures is a stable phase due to the combination of time-reversal symmetry with twofold rotation symmetry^11^. On the other hand, in HgTe/CdTe QWs grown along [111] crystallographic direction, the CB and HH states transform according to different irreducible representations^8^, leading to the subband crossing at *k*_⊥_ = 0. Therefore, in these QWs, one can expect an appearance of an unstable DSM, which is induced by the crystal symmetry of the nanostructure, but is not protected against perturbations. It differs from the symmetry protected 2D DSMs, in which the Dirac points appear at the boundary of the BZ, in the systems with nonsymmorphic symmetries^12^.

In this work, we demonstrate that the TPT in a QW system built from materials with different signs of the effective spin-orbit coupling (SOC) can be mediated by the WSM and the DSM, which opens a unique possibility to induce the transition between these two gapless states without changing the system symmetry or the reordering of the valence subbands. In such a case, the DSM can appear during the TPT, though the CB and VB states at *k*_⊥_ = 0 transform according to the same irreducible representations. We study InGaN-based QWs, for which the TI state can be reached thanks to the large built-in electric field originating from the piezoelectric effect and the spontaneous polarization^13–16^. The idea of using the external or built-in electric field to transform the nontopological QW system to topological one was initially proposed for HgCdTe/CdTe and InAs/GaSb/AlSb QWs and recently, it has been extended to InGaN/GaN, Ge/GaAs and InSb/CdTe quantum heterostructures^13–20^. Although wurtzite group-III nitrides are technologically important semiconductors, the issue of the SOC in these materials is still under scientific debate. For many years, the sequence of the valence bands in wurtzite GaN and InN was believed to be the same, namely the HH band with the Γ_9_ symmetry is above two Γ_7_ bands termed as the light hole (LH) and the crystal field split-off bands. This ordering of the valence bands corresponds to the positive SOC, determined by the positive values of two SOC parameters ${{\rm{\Delta }}}_{so}^{||}$ and ${{\rm{\Delta }}}_{so}^{\perp }$, which are referred to as the SOC constants along the ***c*** axis of the wurtzite lattice and in the plane perpendicular to the ***c*** axis, respectively. The reported values of ${{\rm{\Delta }}}_{so}^{||}$ and ${{\rm{\Delta }}}_{so}^{\perp }$ in InN and GaN, obtained from *ab-initio* band structure calculations and experiments, were in the range from 1 to 25 meV^21–23^. The positive SOC in GaN and InN was taken into account so far in the study of the TPT in InN/GaN and InGaN/GaN QWs^13–15^. In such a case, the TPT is mediated by the DSM arising from the crossing of the HH and CB subbands at *k*_⊥_ = 0^14^. The 2D bulk energy gap (*E*_2*Dg*_) in the TI state was found to reach about 5 meV^14,15^ and 10 meV^13^. These significant values of *E*_2*Dg*_ allow experimental verification of the edge state transport in InN/GaN and InGaN/GaN QWs and also motivate design of new topological devices based on these structures^24,25^. However, recent state of the art *ab-initio* calculations, performed using the quasiparticle self-consistent GW method, have shown that the effective SOC in InN is negative with ${{\rm{\Delta }}}_{so}^{||}$ and ${{\rm{\Delta }}}_{so}^{\perp }$ equal to −9.5 and −5.9 meV, respectively^26^. Consequently, the ordering of the valence bands in InN is anomalous with the LH Γ_7_ band above the HH Γ_9_ band^26^. The negative SOC was also found in zinc-blende HgS and in wurtzite ZnO and TlN^27–29^. TlN has additionally inversion of the CB and VB states in the center of the BZ, which makes it a unique 3D TI with the negative SOC^29^.

**Figure 1:**
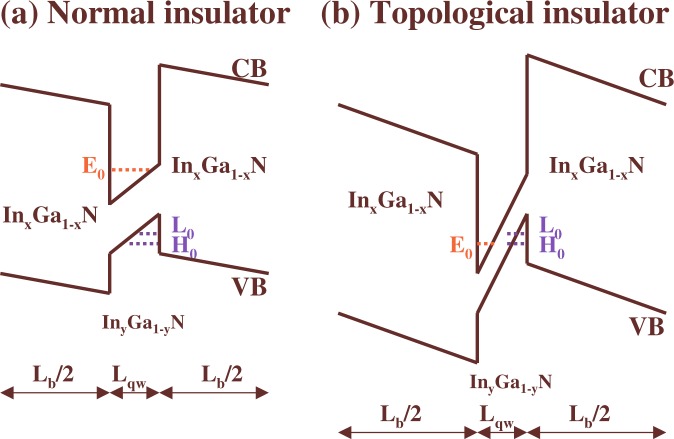
Schematic representation of InGaN-based QWs in the NI (**a**) and TI (**b**) phases. The thickness of In_*y*_Ga_1−y_N QWs is denoted by *L*_*qw*_ and *L*_*b*_ is the width of In_*x*_Ga_1−x_N barriers. The energy levels *E*_0_, *L*_0_ and *H*_0_ correspond to the lowest CB level and the highest LH and HH levels, respectively.

Here, we investigate the influence of the negative SOC on the TPT and the properties of the TI state in InGaN-based QWs grown along the ***c*** axis (see Fig. 1). We employ the realistic eight-band ***k·p*** method, which includes relativistic and nonrelativistic linear in ***k*** terms (see the Methods section). Our calculations show that the negative SOC in InN is not an obstacle to induce the TI phase in InN/InGaN and InGaN/GaN QWs. The *E*_2*Dg*_ in the TI state can reach 2 meV, which enables detection of edge state transport in reasonable experimental conditions. The TPT occurs due to the band inversion between the highest LH subband and the lowest CB subband, and almost always is mediated by the WSM due to the anticrossing of the CB and LH levels at *k*_⊥_ = 0. However, for certain InGaN/GaN QWs, we find that this level anticrossing vanishes, leading to the appearance of the DSM at the boundary between the NI and TI phases. Thus, we reveal a novel transition between the WSM and the DSM and show that it originates from vanishing of the average ${{\rm{\Delta }}}_{so}^{\perp }$ parameter over a QW structure.

## Results and Discussion

**Figure 2:**
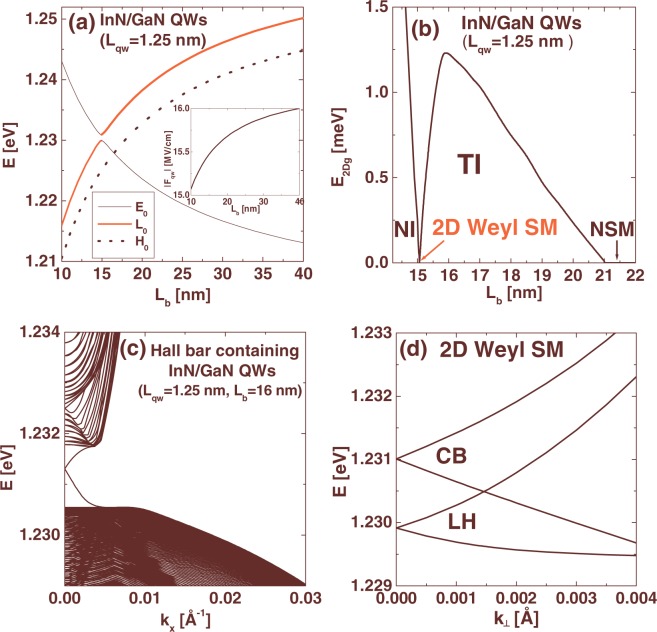
(**a**,**b**) The energy levels *E*_0_, *L*_0_, and *H*_0_ (**a**), and the *E*_2*Dg*_ (**b**) in InN/GaN QWs with *L*_*qw*_ = 1.25 nm as a function of *L*_*b*_. The inset in (**a**) shows the amplitude of the built-in electric field in wells versus *L*_*b*_. (**c**) The dispersion of electronic states in a 1000 nm wide Hall bar structure containing InN/GaN QWs with *L*_*qw*_ = 1.25 nm and *L*_*b*_ = 16 nm. (**d**) The CB and LH subbands in the WSM phase for InN/GaN QWs with *L*_*qw*_ = 1.25 nm and *L*_*b*_ = 15.06 nm.

We consider first InN/GaN multi-QWs with the QW width, *L*_*qw*_ = 1.25 nm, corresponding to 4 monolayers of InN, for which the band structure can be inverted by the built-in electric field^13^. A disadvantage of these nanostructures is significant internal strain, which arises from large lattice misfit between GaN and InN, and causes difficulties in pseudomorphic growth of sufficiently thick wells^30^. In Fig. 2(a), we present the energy levels at *k*_⊥_ = 0 for the lowest CB subband (*E*_0_), the highest LH subband (*L*_0_) and the highest HH subband (*H*_0_) as a function of the barrier thickness, *L*_*b*_. The inset shows the amplitude of the built-in electric field in wells (|*F*_*qw*_|) versus *L*_*b*_. Note that increasing *L*_*b*_ results in increase of |*F*_*qw*_|, according to the well-known formula ${F}_{qw}=\frac{{L}_{b}({P}_{b}-{P}_{qw})}{{L}_{qw}{\lambda }_{b}+{L}_{b}{\lambda }_{qw}}$, where *P*_*qw*_ and *P*_*b*_ denote the polarization in wells and barriers and *λ* is the electric permittivity^13–15^. As a consequence, the *E*_0_ level decreases and the *L*_0_ and *H*_0_ levels increase with increasing *L*_*b*_ in accordance with the quantum confined Stark effect^13–15^. Due to the negative SOC in InN, the *L*_0_ level is above the *H*_0_ level. For *L*_*b*_ near 15 nm, we observe the anticrossing between the *E*_0_ and *L*_0_ levels, since both states transform according to the Γ_7_ irreducible representations. In Fig. 2(b), we show the *E*_2*Dg*_ as a function of *L*_*b*_. One can see that the *E*_2*Dg*_ closes first for *L*_*b*_ = 15.06 nm due to the TPT originating from the inversion of the CB and LH subbands. Then, the *E*_2*Dg*_ vanishes for *L*_*b*_ larger than 21 nm due to the transition from the TI phase to the nonlocal semimetal (NSM) phase, arising from nonlocal overlapping between the LH and HH subbands^14^. The largest value of the *E*_2*Dg*_ in the TI state is about 1.25 meV, which is a few times smaller than it was predicted assuming the positive SOC in InN^13–15^. Nevertheless, the obtained values of the *E*_2*Dg*_ are still large enough to allow experimental verification of the edge state transport in InN/GaN QWs. Here, we would like to mention that the TI state with the *E*_2*Dg*_ of about 1–3 meV has recently been found experimentally in HgTe/CdHgTe QWs^31^. In order to confirm the TPT in InN/GaN QWs, we compute electronic states in a Hall bar represented by a strip structure with the width of 1000 nm. The Hall bar contains InN/GaN QWs with *L*_*qw*_ = 1.25 nm and *L*_*b*_ = 16 nm, which are in the TI state. Figure 2(c) shows the dispersion of electronic states obtained using the 2D effective Hamiltonian, which has been described in the Methods section. One can see metallic edge states arising from the quantum spin Hall effect in the 2D TIs^1^. Unlike the previous studies^13–15^, we find that the Dirac point of the edge states dispersion curve is located near the middle of the *E*_2*Dg*_, due to the anomalous ordering of the LH and HH subbands, originating from the negative SOC in InN. Now, we focus on the WSM phase occurring when the system goes through the TPT. In Fig. 2(d), we present the CB and LH subbands in InN/GaN QWs with *L*_*qw*_ = 1.25 nm and *L*_*b*_ = 15.06 nm. The obtained WSM can be described by the critical magnitude of the in-plane wave vector *k*_0_ = 0.0015 Å^−1^ and the anticrossing of the energy levels Δ_0_ = *E*_0_−*L*_0_ equal to 1.1 meV.

**Figure 3:**
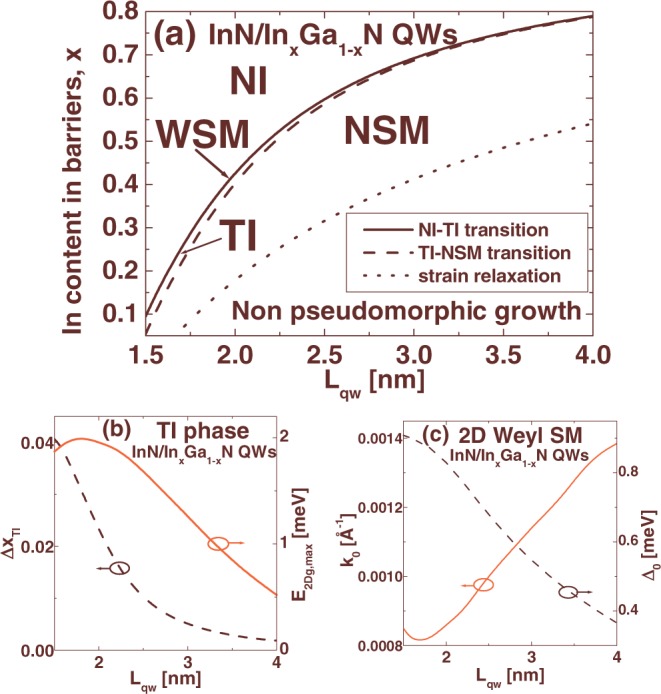
(**a**) The phase diagram illustrating four phases, i.e., the NI, the WSM, the TI, and the NSM, in InN/In_*x*_Ga_1−*x*_N QWs with *L*_*b*_ = 40 nm. Dotted line corresponds to the critical In content in the barriers, *x*_*c*_, above which pseudomorphic growth is possible. (**b**) The Δ*x*_*TI*_ (left axis) and the ${E}_{2Dg,\max }$ (right axis) in InN/In_*x*_Ga_1−*x*_N QWs as a function of *L*_*qw*_. (**c**) The parameters *k*_0_ (left axis) and Δ_0_ (right axis) for the WSM as a function of *L*_*qw*_.

We extend our study to InN/In_*x*_Ga_1−*x*_N QWs. We consider multi-QW structures pseudomorphically grown on unstrained In_*x*_Ga_1−*x*_N buffer layers, acting as virtual substrates. In these structures, the magnitude of internal strain decreases linearly with increasing the In content in barriers, which significantly facilitates pseudomorphic growth. It is worth mentioning that In_*x*_Ga_1−*x*_N layers can be grown over the entire composition range and they have already been used as virtual substrates in the growth of In_*y*_Ga_1−*y*_N/In_*x*_Ga_1−*x*_N QWs for optoelectronic applications^32,33^. We focus on multi-QWs with wide barriers (similarly to refs^13–15^, we take *L*_*b*_ = 40 nm), since in these structures, |*F*_*qw*_| is much larger than the amplitude of the built-in electric field in the barriers (|*F*_*b*_| = |*F*_*qw*_|*L*_*qw*_/*L*_*b*_) and the TPT can be more easily obtained^13–15^. Note that the dependence of |*F*_*qw*_| on *L*_*b*_ is rather weak for large *L*_*b*_ and thus, we study the TPT in InN/In_*x*_Ga_1−*x*_N QWs as a function of *L*_*qw*_ and In content in the barriers, for fixed *L*_*b*_. In Fig. 3(a), we show a phase diagram illustrating four phases, i.e., the NI, the WSM, the TI, and the NSM, for InN/In_*x*_Ga_1−*x*_N QWs with *L*_*qw*_ in the range from 1.5 to 4 nm. Dotted line represents the critical In content in the barriers of InN/In_*x*_Ga_1−*x*_N heterostructures, *x*_*c*_, above which pseudomorphic growth without strain relaxation is possible. The dependence of *x*_*c*_ on *L*_*qw*_ is obtained using a modification of the model of Fischer-Kühne-Richter^34^. For each value of *L*_*qw*_, all four phases can be achieved in pseudomorphic InN/In_*x*_Ga_1−*x*_N QWs by properly choosing the composition of the barriers. Note, that the distance between the solid and dotted lines increases with increasing *L*_*qw*_, which indicates that the band inversion can be obtained easier in wider QWs with larger In content in the barriers. However, the properties of the TI state deteriorate with increasing *L*_*qw*_, as we demonstrate in Fig. 3(b). For each *L*_*qw*_, the TI phase can be characterized by the window of In content within which the TI state is achieved, Δ*x*_*TI*_, and by the largest value of the *E*_2*Dg*_, which is denoted by ${E}_{2Dg,\max }$. In Fig. 3(b), we show the Δ*x*_*TI*_ (left axis) and the ${E}_{2Dg,\max }$ (right axis) as a function of *L*_*qw*_. One can see that the Δ*x*_*TI*_ decreases with increasing *L*_*qw*_ and the ${E}_{2Dg,\max }$ reaches the largest value of 2 meV for relatively narrow QWs with *L*_*qw*_ = 1.8 nm. The TI state with the ${E}_{2Dg,\max }$ = 2 meV is found for 1.8 nm wide InN/In_0.316_Ga_0.684_N QWs. In Fig. 3(c), we present the parameters *k*_0_ (left axis) and Δ_0_ (right axis) for the WSM in InN/In_*x*_Ga_1−*x*_N QWs as a function of *L*_*qw*_. One can see that both *k*_0_ and Δ_0_ are significant and show no tendency to vanish simultaneously, which indicates that in InN/In_*x*_Ga_1−*x*_N QWs, we deal solely with the WSM between the NI and TI phases.

**Figure 4:**
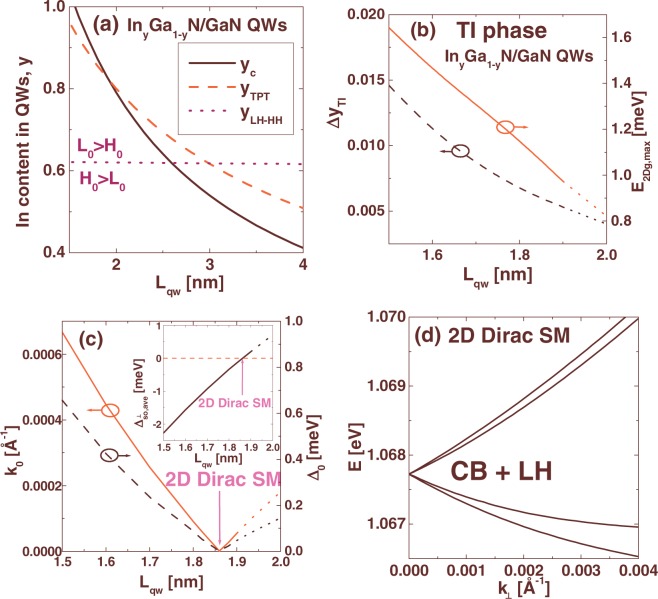
(**a**) In content in In_*y*_Ga_1−*y*_N/GaN QWs, for which the TPT occurs (dashed line) and the critical In content for pseudomorphic growth, *y*_*c*_ (solid line), as a function of *L*_*qw*_. Above the dotted line, the highest LH subband has higher energy than the highest HH subband. (**b**) The values of Δ*y*_*TI*_ (left axis) and ${E}_{2Dg,\max }$ (right axis) for the TI phase in In_*y*_Ga_1−*y*_N/GaN QWs as a function of *L*_*qw*_. (**c**) The parameters *k*_0_ (left axis) and Δ_0_ (right axis) for the WSM as a function of *L*_*qw*_. The inset shows the average ${{\rm{\Delta }}}_{so}^{\perp }$ parameter over a QW structure at the *E*_0_ and *L*_0_ states as a function of *L*_*qw*_. The dotted lines in (**b**) and (**c**) represent the QW structures, for which the In content is larger than *y*_*c*_. (**d**) The CB and LH subbands in the DSM state for In_0.838_Ga_0.162_N/GaN QWs with *L*_*qw*_ = 1.86 nm and *L*_*b*_ = 40 nm.

Finally, we consider In_*y*_Ga_1−*y*_N/GaN multi-QWs with wide barriers (*L*_*b*_ = 40 nm), which are grown on GaN substrate. Figure 4(a) shows the In content, for which the TPT occurs (dashed line) and the critical In content for pseudomorphic growth *y*_*c*_, obtained using the Fischer-Kühne-Richter model (solid line)^34^, as a function of *L*_*qw*_. We find that the TPT can be achieved only in the QWs with *L*_*qw*_ smaller than 1.9 nm, for which pseudomorphic growth is possible. The dotted line separates the QWs, for which the highest LH subband is above the highest HH subband (*L*_0_ > *H*_0_), from those QWs having the opposite ordering of the valence subbands (*H*_0_ > *L*_0_). Above the dotted line, we have *L*_0_ > *H*_0_ and thus, in pseudomorphically grown In_*y*_Ga_1−*y*_N/GaN QWs, the TPT occurs always due to inversion of the highest LH subband and the lowest CB subband. In Fig. 4(b), we show the window of In content within which the TI state is achieved, Δ*y*_*TI*_ (left axis), and the ${E}_{2Dg,\max }$ (right axis) as a function of *L*_*qw*_. Both these parameters decrease with increasing *L*_*qw*_, which indicates that the properties of the TI state deteriorate for wider In_*y*_Ga_1−*y*_N/GaN QWs. The dotted lines in Figs 4(b,c) correspond to the QW structures, for which the In content is larger than *y*_*c*_, and thus, non-pseudomorphic growth with partial strain relaxation can occur. Figure 4(c) presents the parameters *k*_0_ (left axis) and Δ_0_ (right axis) for the WSM occurring when the system goes through the TPT. Interestingly, both *k*_0_ and Δ_0_ vanish for *L*_*qw*_ = 1.86 nm, which indicates that in this particular case, the TPT is mediated by the DSM instead of the WSM. Indeed, as shown in Fig. 4(d), for In_0.838_Ga_0.162_N/GaN QWs with *L*_*qw*_ = 1.86 nm, we obtain a crossing of the *E*_0_ and *L*_0_ levels that leads to the appearance of the DSM. In order to find an explanation of this effect, we compute the average ${{\rm{\Delta }}}_{so}^{\perp }$ parameter over a QW structure at the LH and CB states for *k*_⊥_ = 0. Note that ${{\rm{\Delta }}}_{so}^{\perp }$ couples different spins in the valence band states and determines the coupling between the LH and CB states at *k*_⊥_ = 0 (see the Methods section). The results are presented in the inset in Fig. 4(c). One can see that the average ${{\rm{\Delta }}}_{so}^{\perp }$ vanishes exactly for the same QW structure, for which the DSM appears. Vanishing of the average ${{\rm{\Delta }}}_{so}^{\perp }$ is possible, since the ${{\rm{\Delta }}}_{so}^{\perp }$ parameter has different signs in wells (${{\rm{\Delta }}}_{so}^{\perp }$ = −2.32 meV) and barriers (${{\rm{\Delta }}}_{so}^{\perp }$ = 16.2 meV). Importantly, the observed transition between the WSM and the DSM occurs without changing the crystal symmetry of QWs or the reordering of the valence subbands. This new phenomenon can be verified by several experimental methods including magnetotransport experiments and THz radiation absorption measurements, which have recently been proposed to study the gapless states in HgTe/CdTe QWs^9^.

To conclude, we have demonstrated that in InN/In_*x*_Ga_1−*x*_N and In_*y*_Ga_1−*y*_N/GaN QWs, the negative SOC is not an obstacle to induce the TI state with the significant *E*_2*Dg*_, enabling measurements of edge state transport in reasonable conditions. The magnitude of *E*_2*Dg*_ decreases with increasing *L*_*qw*_ in In_*y*_Ga_1−*y*_N/GaN QWs, whereas in InN/In_*x*_Ga_1−*x*_N QWs, it shows a non-monotonic dependence on *L*_*qw*_, with the largest value of 2 meV reached for 1.8 nm wide InN/In_0.316_Ga_0.684_N QWs. The TPT in InGaN-based QWs is usually mediated by the WSM arising from the anticrossing of the CB and LH levels at *k*_⊥_ = 0. However, for certain In_*y*_Ga_1−*y*_N/GaN QWs, we have found that this anticrossing vanishes, leading to the appearance of the DSM. The novel transition between the WSM and the DSM occurs due to vanishing of the average ${{\rm{\Delta }}}_{so}^{\perp }$ parameter in the QW system. This effect originates from opposite signs of the ${{\rm{\Delta }}}_{so}^{\perp }$ parameter in wells and barriers without changing the system symmetry or the reordering of the valence subbands. Thus, our work reveals that InGaN-based QWs with inverted bands represent a unique topological QW system, which differs significantly from the conventional 2D TIs based on HgTe/CdTe and InAs/GaSb/AlSb QWs. We hope that these results will stimulate intensive theoretical and experimental studies towards fabrication and investigation of group-III nitride topological materials and devices. Since the negative SOC occurs also in HgS and TlN, the similar effects to these observed in InGaN-based QWs may appear in other 2D topological nanostructures.

## Methods

In this section, we provide the details on the eight-band ***k·p*** method, which we apply to calculate the subband dispersion in InGaN-based QWs. Our approach includes relativistic and nonrelativistic linear-***k*** terms, which play a significant role in accurate determination of the valence bands of GaN and InN^26^. We also determine the effective 2D Hamiltonian, which is used to calculate the electronic states in a Hall bar containing InN/GaN QWs.

According to refs^26,35^, the 6 × 6 valence band ***k·p*** Hamiltonian for unstrained wurtzite semiconductors can be written as

1 $${{\rm H}}^{6\times 6}={{\rm{\Delta }}}_{1}{J}_{z}^{2}+{{\rm{\Delta }}}_{2}{J}_{z}{\sigma }_{z}+\sqrt{2}{{\rm{\Delta }}}_{3}({J}_{+}{\sigma }_{-}+{J}_{-}{\sigma }_{+})+({A}_{1}+{A}_{3}{J}_{z}^{2}){k}_{z}^{2}+({A}_{2}+{A}_{4}{J}_{z}^{2}){k}_{\perp }^{2}-{A}_{5}({J}_{+}^{2}{k}_{-}^{2}+{J}_{-}^{2}{k}_{+}^{2})-2i{A}_{6}{k}_{z}(\{{J}_{z},{J}_{+}\}{k}_{-}-\{{J}_{z},{J}_{-}\}{k}_{+})+{A}_{7}({k}_{-}{J}_{+}+{k}_{+}{J}_{-})+({\alpha }_{1}+{\alpha }_{3}{J}_{z}^{2})({\sigma }_{+}{k}_{-}+{\sigma }_{-}{k}_{+})+{\alpha }_{2}({J}_{+}^{2}{\sigma }_{-}{k}_{-}+{J}_{-}^{2}{\sigma }_{+}{k}_{+})+2{\alpha }_{4}{\sigma }_{z}(\{{J}_{z},{J}_{+}\}{k}_{-}-\{{J}_{z},{J}_{-}\}{k}_{+})+2i{\alpha }_{5}{k}_{z}(\{{J}_{z},{J}_{+}\}{\sigma }_{-}-\{{J}_{z},{J}_{-}\}{\sigma }_{+})$$

where ${J}_{\pm }=\frac{1}{\sqrt{2}}(\,\pm \,i{J}_{x}-{J}_{y})$, ${\sigma }_{\pm }=\frac{1}{\sqrt{2}}(\,\pm \,i{\sigma }_{x}-{\sigma }_{y})$, $\{{J}_{z},{J}_{+}\}=\frac{1}{2}({J}_{z}{J}_{+}+{J}_{+}{J}_{z})$, *k*_±_ = *k*_*x*_±*ik*_*y*_, ${k}_{\perp }^{2}={k}_{x}^{2}+{k}_{y}^{2}$. The parameters Δ_1_, ${{\rm{\Delta }}}_{2}={{\rm{\Delta }}}_{so}^{||}/3$, and ${{\rm{\Delta }}}_{3}={{\rm{\Delta }}}_{so}^{\perp }/3$ are the crystal field splitting and the spin-orbit coupling splittings, respectively. The parameters *A*_1_, …, *A*_6_ are inverse effective-mass type parameters, the *A*_7_ coefficient determines a nonrelativistic linear-***k*** term and the parameters *α*_1_, …, *α*_5_ describe the relativistic (spin-dependent) terms linear in ***k***. The *σ*_*x*_, *σ*_*y*_, and *σ*_*z*_ denote the Pauli spin matrices and the *J*_*x*_, *J*_*y*_, and *J*_*z*_ are the angular momentum matrices:

$$\begin{array}{ccc}{J}_{x}=\frac{1}{\sqrt{2}}[\begin{array}{ccc}0 & 1 & 0\\ 1 & 0 & 1\\ 0 & 1 & 0\end{array}], & {J}_{y}=\frac{i}{\sqrt{2}}[\begin{array}{ccc}0 & -1 & 0\\ 1 & 0 & -1\\ 0 & 1 & 0\end{array}], & {J}_{z}=[\begin{array}{ccc}1 & 0 & 0\\ 0 & 0 & 0\\ 0 & 0 & -1\end{array}]\end{array}.$$

The Hamiltonian Η^6×6^ can be represented in a matrix form as follows:

2 $${{\rm H}}^{6\times 6}=[\begin{array}{cccccc}F & {K}^{\ast } & {M}_{-}^{\ast } & 0 & -{W}^{\ast } & 0\\ K & G & -{N}_{+} & -{W}^{\ast } & -T & {\rm{\Delta }}\\ {M}_{-} & -{N}_{+}^{\ast } & L & 0 & {{\rm{\Delta }}}^{\ast } & -{S}^{\ast }\\ 0 & -W & 0 & F & K & -{M}_{+}\\ -W & -{T}^{\ast } & {\rm{\Delta }} & {K}^{\ast } & G & {N}_{-}^{\ast }\\ 0 & {{\rm{\Delta }}}^{\ast } & -S & -{M}_{+}^{\ast } & {N}_{-} & L\end{array}]\begin{array}{c}|-(X+iY)/\sqrt{2},\uparrow \rangle \\ |(X-iY)/\sqrt{2},\uparrow \rangle \\ |Z,\uparrow \rangle \\ |(X-iY)/\sqrt{2},\downarrow \rangle \\ |-(X+iY)/\sqrt{2},\downarrow \rangle \\ |Z,\downarrow \rangle \end{array},$$

where $F={{\rm{\Delta }}}_{1}+{{\rm{\Delta }}}_{2}+({A}_{2}+{A}_{4}){k}_{\perp }^{2}+({A}_{1}+{A}_{3}){k}_{z}^{2}$, *G* = *F*−2Δ_2_, $L={A}_{2}{k}_{\perp }^{2}+{A}_{1}{k}_{z}^{2}$, $K={A}_{5}{k}_{+}^{2}$, *M*_+_ = [*A*_6_*k*_*z*_ + *i*(*A*_7_ + *α*_4_)]*k*_+_, *M*_−_ = [*A*_6_*k*_*z*_ − *i*(*A*_7_ + *α*_4_)]*k*_+_, *N*_+_ = [*A*_6_*k*_*z*_ + *i*(*A*_7_ − *α*_4_)]*k*_+_, *N*_−_ = [*A*_6_*k*_*z*_ − *i*(*A*_7_ − *α*_4_)]*k*_+_, ${\rm{\Delta }}=\sqrt{2}{{\rm{\Delta }}}_{3}+i{\alpha }_{5}{k}_{z}$, *S* = *iα*_1_*k*_+_, *T* = *iα*_2_*k*_+_, and *W* = *i*(*α*_1_ + *α*_3_)*k*_+_.

The above Hamiltonian can be reduced to the ***k·p*** Hamiltonians presented in refs^36,37^ by neglecting the relativistic and nonrelativistic linear-***k*** terms. Here, we note that the coefficients *A*_5_ and *A*_6_ have opposite signs to those in refs^36,37^. (The difference in sign of the *A*_5_ and *A*_6_ coefficients was also discussed in refs^38,39^) In order to take into account the coupling between the conduction band and the valence bands, we enlarge the Hamiltonian (2) to the eight-band model using the approach applied in refs^13,14^. We reduce the parameter Δ to $\sqrt{2}{{\rm{\Delta }}}_{3}$, since *α*_5_ = 0 for GaN and InN^26^. The resulting H^8×8^ Hamiltonian has the form

3 $${{\rm{{\rm H}}}}^{8\times 8}=[\begin{array}{cccccccc}{H}_{c} & -Q & {Q}^{\ast } & R & 0 & 0 & 0 & 0\\ -{Q}^{\ast } & F & {K}^{\ast } & {M}_{-}^{\ast } & 0 & 0 & -{W}^{\ast } & 0\\ Q & K & G & -{N}_{+} & 0 & -{W}^{\ast } & -T & \sqrt{2}{{\rm{\Delta }}}_{3}\\ R & {M}_{-} & -{N}_{+}^{\ast } & L & 0 & 0 & \sqrt{2}{{\rm{\Delta }}}_{3} & -{S}^{\ast }\\ 0 & 0 & 0 & 0 & {H}_{c} & {Q}^{\ast } & -Q & R\\ 0 & 0 & -W & 0 & Q & F & K & -{M}_{+}\\ 0 & -W & -{T}^{\ast } & \sqrt{2}{{\rm{\Delta }}}_{3} & -{Q}^{\ast } & {K}^{\ast } & G & {N}_{-}^{\ast }\\ 0 & 0 & \sqrt{2}{{\rm{\Delta }}}_{3} & -S & R & -{M}_{+}^{\ast } & {N}_{-} & L\end{array}]\begin{array}{c}|iS,\uparrow \rangle \\ |-(X+iY)/\sqrt{2},\uparrow \rangle \\ |(X-iY)/\sqrt{2},\uparrow \rangle \\ |Z,\uparrow \rangle \\ |iS,\downarrow \rangle \\ |(X-iY)/\sqrt{2},\downarrow \rangle \\ |-(X+iY)/\sqrt{2},\downarrow \rangle \\ |Z,\downarrow \rangle \end{array}$$

where ${H}_{c}={E}_{vb}+{E}_{g}+{A}_{c\perp }{k}_{\perp }^{2}+{A}_{c||}{k}_{z}^{2}$, $Q={P}_{2}{k}_{+}/\sqrt{2}$, and *R* = *P*_1_*k*_*z*_. The top valence band energy and the energy gap are denoted by *E*_*vb*_ and *E*_*g*_, respectively, *A*_*c*⊥_ and *A*_*c*||_ describe the dispersion of the conduction band, whereas *P*_1_ and *P*_2_ are the Kane parameters^37^. The parameters *A*_1_, …, *A*_6_ occurring in the valence band part of the Hamiltonian H^8×8^ have to be rescaled according to ref.^40^.

In order to determine the electronic states in a QW grown along [0001] crystallographic direction of the wurtzite structure, we include the strain effects and the built-in electric field originating from the piezoelectric effect and the spontaneous polarization^14,15^. Due to large lattice misfit between GaN and InN, we calculate strain and the built-in electric field taking into account the effects of nonlinear elasticity and nonlinear piezoelectricity, according to refs^14,15,41^. Then, replacing *k*_*z*_ in the Hamiltonian H^8×8^ by the operator $-i\frac{\partial }{\partial z}$, we get the eight-band Schrödinger-type equation,

4 $$\sum _{\beta =1}^{8}{{\rm{{\rm H}}}}_{\alpha ,\beta }^{8\times 8}({\overrightarrow{k}}_{\perp },{k}_{z}=-\,i\frac{\partial }{\partial z}){F}_{m,\beta }(z,{\overrightarrow{k}}_{\perp })={E}_{m}({\overrightarrow{k}}_{\perp }){F}_{m,\alpha }(z,{\overrightarrow{k}}_{\perp }),\,\alpha =1,\mathrm{...},8,$$

where ${E}_{m}({\overrightarrow{k}}_{\perp })$ and ${F}_{m,\beta }(z,{\overrightarrow{k}}_{\perp })$ are the energies and the envelope functions of the QW states. Since in QW heterostructures, the material parameters depend on position, we use the standard symmetrization to ensure the Hermiticity of operators containing the product of functions and derivatives^14^. The subband dispersion in InN/GaN, InN/InGaN, and InGaN/GaN QWs is obtained by solving numerically Eq. (4)^14^.

To calculate the electronic states in a Hall bar structure of finite width, represented by infinitely long strip in the QW plane, we focus on the lowest CB subband and the highest LH and HH subbands. For these subbands, we generate the 6 × 6 effective 2D Hamiltonian, using the mini-band **k·p** method and the Löwdin perturbation approach^13,14^. In the first step, we define the *z*-direction averaged Hamiltonian

5 $${{\rm{{\rm H}}}}_{m,n}=\langle {{\rm{\Psi }}}_{m,{\overrightarrow{k}}_{\perp }}|{{\rm{{\rm H}}}}^{8\times 8}({\overrightarrow{k}}_{\perp },{k}_{z}=-i\frac{\partial }{\partial z})|{{\rm{\Psi }}}_{n,{\overrightarrow{k}}_{\perp }}\rangle =\sum _{\alpha ,\beta =1}^{8}\int dz{F}_{m,\alpha }^{\ast }(z){{\rm{{\rm H}}}}_{\alpha ,\beta }^{8\times 8}{F}_{n,\beta }(z),$$

where ${{\rm{\Psi }}}_{m,{\overrightarrow{k}}_{\perp }}={e}^{i{\overrightarrow{k}}_{\perp }{\overrightarrow{r}}_{\perp }}{[{F}_{m,1}(z),\mathrm{...},{F}_{m,8}(z)]}^{T}$ is the Luttinger-Kohn basis set for the mini-band **k·p** method in the vicinity of the Γ point, and *F*_*m*,*α*_(*z*) = *F*_*m*,*α*_(*z*,${\overrightarrow{k}}_{\perp }$ = 0). This Hamiltonian can be divided into two parts ${{\rm{{\rm H}}}}_{m,n}={{\rm{{\rm H}}}}_{m,n}^{0}+{{\rm{{\rm H}}}}_{m,n}^{^{\prime} }$, where

6 $${{\rm{{\rm H}}}}_{m,n}^{0}=\langle {{\rm{\Psi }}}_{m,{\overrightarrow{k}}_{\perp }}|{{\rm{{\rm H}}}}^{8\times 8}({\overrightarrow{k}}_{\perp }=0,{k}_{z}=-\,i\frac{\partial }{\partial z})|{{\rm{\Psi }}}_{n,{\overrightarrow{k}}_{\perp }}\rangle ={\delta }_{m,n}{E}_{m}({\overrightarrow{k}}_{\perp }=0),$$

and

7 $${{\rm{{\rm H}}}}_{m,n}^{^{\prime} }=\langle {{\rm{\Psi }}}_{m,{\overrightarrow{k}}_{\perp }}|{{\rm{{\rm H}}}^{\prime} }^{8\times 8}({\overrightarrow{k}}_{\perp },{k}_{z}=-\,i\frac{\partial }{\partial z})|{{\rm{\Psi }}}_{n,{\overrightarrow{k}}_{\perp }}\rangle =\sum _{\alpha ,\beta =1}^{8}\int dz{F}_{m,\alpha }^{\ast }(z){\rm{{\rm H}}}{^{\prime} }_{\alpha ,\beta }^{8\times 8}{F}_{n,\beta }(z).$$

Then, we consider six eigenstates of the Hamiltonian ${{\rm{{\rm H}}}}_{m,n}^{0}$: $|E,{j}_{z}=\pm 1/2\rangle$, $|LH,{j}_{z}=\pm 1/2\rangle$, and $|HH,{j}_{z}=\pm 3/2\rangle$, corresponding to the lowest CB subband and the highest LH and HH subbands, respectively. The symbol *j*_*z*_ denotes the projection of the total angular momentum on the *z*-axis. The coupling between the six states $|E,{j}_{z}=\pm 1/2\rangle$, $|LH,{j}_{z}=\pm 1/2\rangle$, $|HH,{j}_{z}=\pm 3/2\rangle$ and the rest of the eigenstates of the Hamiltonian ${{\rm{{\rm H}}}}_{m,n}^{0}$ can be eliminated to the second order in ***k*** using the Löwdin perturbation method^13,14^. For practical implementation, we consider additional 114 states of the Hamiltonian ${{\rm{{\rm H}}}}_{m,n}^{0}$ corresponding to 19 double degenerated levels for the CB, LH and HH subbands. The effective Hamiltonian has the following form:

8 $${{\rm{{\rm H}}}}_{eff}=[\begin{array}{cccccc}{E}_{0}+{E}_{1}{k}_{\perp }^{2} & {C}_{2}{k}_{-} & {C}_{1}{k}_{+} & i{C}_{3}{k}_{-} & iM{k}_{\perp }^{2} & i{B}_{2}{k}_{-}^{2}\\ {C}_{2}{k}_{+} & {L}_{0}+{L}_{1}{k}_{\perp }^{2} & {B}_{1}{k}_{+}^{2} & -iM{k}_{\perp }^{2} & i{C}_{4}{k}_{+} & i{C}_{5}{k}_{-}\\ {C}_{1}{k}_{-} & {B}_{1}{k}_{-}^{2} & {H}_{0}+{H}_{1}{k}_{\perp }^{2} & -i{B}_{2}{k}_{-}^{2} & i{C}_{5}{k}_{-} & 0\\ -i{C}_{3}{k}_{+} & iM{k}_{\perp }^{2} & i{B}_{2}{k}_{+}^{2} & {E}_{0}+{E}_{1}{k}_{\perp }^{2} & -{C}_{2}{k}_{+} & -{C}_{1}{k}_{-}\\ -iM{k}_{\perp }^{2} & -i{C}_{4}{k}_{-} & -i{C}_{5}{k}_{+} & -{C}_{2}{k}_{-} & {L}_{0}+{L}_{1}{k}_{\perp }^{2} & {B}_{1}{k}_{-}^{2}\\ -i{B}_{2}{k}_{+}^{2} & -i{C}_{5}{k}_{+} & 0 & -{C}_{1}{k}_{+} & {B}_{1}{k}_{+}^{2} & {H}_{0}+{H}_{1}{k}_{\perp }^{2}\end{array}]\begin{array}{c}|E,1/2\rangle \\ |LH,-1/2\rangle \\ |HH,3/2\rangle \\ |E,-1/2\rangle \\ |LH,1/2\rangle \\ |HH,-3/2\rangle \end{array}$$

where *E*_0_, *L*_0_, and *H*_0_ correspond to the energies of states $|E,{j}_{z}=\pm 1/2\rangle$, $|LH,{j}_{z}=\pm 1/2\rangle$, and $|HH,{j}_{z}=\pm 3/2\rangle$, respectively. The obtained Hamiltonian Η_*eff*_ has the same form to that used in refs^14,15^. The linear-***k*** terms of the Hamiltonian Η^8×8^ with the coefficients *α*_1_, …, *α*_4_ and *A*_7_ do not change the structure of Η_*eff*_, but they contribute significantly to the values of the coefficients *C*_1_, …, *C*_5_.

**Figure 5:**
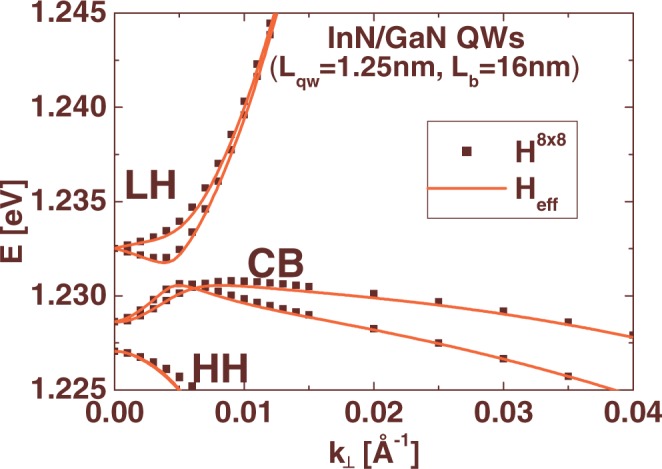
The subband dispersion in InN/GaN QWs with *L*_*qw*_ = 1.25 nm and *L*_*b*_ = 16 nm, obtained using the eight-band **k·p** Hamiltonian, Η^8×8^, (squares) and the effective 2D Hamiltonian, Η_*eff*_ (solid lines).

In Fig. 5, we compare the subband dispersions for an exemplary InN/GaN multi-QW structure (*L*_*qw*_ = 1.25 nm and *L*_*b*_ = 16 nm) obtained using the Η^8×8^ Hamiltonian (squares) and the Hamiltonian Η_*eff*_ (solid lines). We find that the Hamiltonian Η_*eff*_ describes quite well the in-plane dispersion of the lowest CB subband and the highest LH and HH subbands and thus, it can be applied to calculate the electronic states in a Hall bar.

In the calculations of the electronic states in InN/GaN QWs, we use the valence band parameters from ref.^26^ and the deformation potentials from ref.^13^. For InN/InGaN and InGaN/GaN QWs, we apply the valence band parameters from ref.^26^ (assuming linear dependence on composition in InGaN), the deformation potentials from ref.^42^. We determine the spontaneous polarization and the piezoelectric polarization according to ref.^41^. Nonlinear dependence on composition in InGaN is taken into account for the first-order piezoelectric constants, whereas in the case of the spontaneous polarization and the second-order piezoelectric constants, linear dependence on composition is assumed^41^. The second-order elastic constants and the third-order elastic constants (assuming linear dependence on composition in InGaN) are taken from ref.^43^ and ref.^14^, respectively.

## Data Availability

The datasets generated and analysed during the current study are available from the corresponding author on reasonable request.
